# Assessment of *In Vivo* Antidiabetic Properties of Umbelliferone and Lupeol Constituents of Banana (*Musa* sp. var. Nanjangud Rasa Bale) Flower in Hyperglycaemic Rodent Model

**DOI:** 10.1371/journal.pone.0151135

**Published:** 2016-03-22

**Authors:** Ramith Ramu, Prithvi S. Shirahatti, Nanjunda Swamy S., Farhan Zameer, Bhadrapura Lakkappa Dhananjaya, Nagendra Prasad M. N.

**Affiliations:** 1 Department of Biotechnology, Sri Dharmasthala Manjunatheshwara College of Post Graduate Centre, Ujire, Dakshina Kannada – 574240, Karnataka, India; 2 Department of Biotechnology, Sri Jayachamarajendra College of Engineering, JSS Technical Institutions Campus, Mysore – 570 006, Karnataka, India; 3 Department of Studies in Biotechnology, Microbiology and Biochemistry, Mahajana Life Science Research Centre, Pooja Bhagavat Memorial Mahajana PG Centre, Mysore – 570 016, Karnataka, India; 4 Toxinology/Toxicology and Drug Discovery Unit, Centre for Emerging Technologies (CET), Jain Global Campus, Jain University, Kanakapura Taluk, Ramanagara Dist, Karnataka-562112, India; Stellenbosch University, SOUTH AFRICA

## Abstract

Banana is an extensively cultivated plant worldwide, mainly for its fruit, while its ancillary product, the banana flower is consumed as a vegetable and is highly recommended for diabetics in the traditional Indian medicine system. This study is based on an investigation of the *in vivo* antihyperglycaemic activity of Umbelliferone (C1) and Lupeol (C2) isolated from the ethanol extract of banana flower (EF) in alloxan induced diabetic rat model. Diabetic rats which were administered with C1, C2 and EF (100 and 200 mg/kg b. wt.) for 4 weeks showed deterioration in fasting hyperglycaemia and reversal of abnormalities in serum/urine protein, urea and creatinine, when compared to the diabetic control group of rats. The diabetic group of rats fed with EF, C1 and C2 (100 mg/kg b. wt.) once daily, for a period of 28 days resulted in a significant reduction of diabetic symptoms *viz*., polyphagia, polydipsia, polyuria and urine sugar together with an improved body weight. HbA1c extent was reduced whereas levels of insulin and Hb were increased. Both the extract and compounds wielded positive impacts in diabetic rats by reversal of altered activities of hepatic marker enzymes *viz*., aspartate transaminase (AST), alanine transaminase (ALT), alkaline phosphatase (ALP); glycolytic enzyme (hexokinase); shunt enzyme (glucose-6-phosphate dehydrogenase); gluconeogenic enzymes (glucose-6-phosphatase, fructose-1,6-bisphosphatase, lactate dehydrogenase) and pyruvate kinase. The characteristic diabetic complications such as hypercholesterolemia and hypertriacylglycerolemia also significantly reverted to normal in the serum/liver of diabetic rats. Besides these, the treatment increased the activities of enzymatic and non-enzymatic antioxidants in the serum and liver. The histological observations revealed a marked regeneration of the β-cells in the drug treated diabetic rats. In conclusion, the present study illustrates that EF, C1 and C2 enhances the glycolytic activities, besides increasing the hepatic glucose utilization in diabetic rats by stimulating insulin secretion from the remnant β-cells along with potential enzymatic and non-enzymatic antioxidant activities.

## Introduction

Diabetes mellitus is a chronic disorder characterized by hyperglycaemia, elicited due to a disfunctional carbohydrate, lipid and protein metabolism with absolute/relative deficiencies in the insulin secretion or its action [1]. Clinical and preclinical evidences suggest a strong association of diabetes to oxidative stress, which is the resultant of a significantly higher reactive oxygen species (ROS) produced in response to the prolonged hyperglycaemic state. During the pathogenesis of diabetes, a significantly higher generation of the ROS leads to non-enzymatic protein glycosylation, glucose auto-oxidation, impaired antioxidant enzyme and lipid peroxide formation [2]. Further, the lipid peroxidation has a direct implication on the blood lipid profile, which raises the risks associated with other disease conditions like atherosclerosis and cardiovascular disorders, thus suggesting the necessity to control not only the blood glucose levels but also the blood lipid levels for optimum diabetes management [3]. Acute elevation in the blood glucose levels also inhibit hepatic glucose production and instigate the expression of phosphoenol pyruvate carboxykinase enzyme gene which constitutes the key regulatory enzyme of gluconeogenesis [4]. Impaired regulation of these pathways owing to persistent hyperglycaemia leads to several diabetic complications, including oxidative stress to various tissues that are characterized by several biomarkers in the liver [5]. Apart from the elevated levels of these biomarkers, the toxic effects exerted by hyperglycaemia on liver (the chief organ channelising the excessive circulating blood glucose for glycogenesis) is further augmented through lipid peroxidation of the hepatocellular membranes leading to many serious complications in the liver. This cascade of cell necrosis and tissue damage as a result of ROS suggests the requirement of an effective antioxidant therapy for diabetes management [6]. Recent studies have reported amelioration in the liver damage caused by chronic hyperglycaemia after administration of antioxidant adjunct suggesting their potential benefit in management of diabetic complications [7–8].

Traditionally, the dietary interventions derived from natural resources have been identified for its potential in the management of diabetic complications. Though the mode of action is not scientifically explained pertaining to most of the traditional medicinal formulations, their effectiveness in improving the condition have justified their use in Indian traditional system of medicines [9]. In this aspect, banana (belonging to *musaceae* family) flower, generally used as a vegetable in India, is recommended for diabetics in Indian traditional medicine (*Ayurveda*). The earlier work in our laboratory has shown that Umbelliferone and Lupeol, isolated from ethanol extract of banana flower (EF) have significant *in vitro* antihyperglycaemic activity [10]. Hence, the present study was undertaken to investigate the *in vivo* antidiabetic and antioxidant potential of Umbelliferone and Lupeol isolated from EF in an alloxan induced diabetic rats. The work highlights the effects of EF and its isolated compounds (Umbelliferone and Lupeol) on carbohydrate metabolizing enzymes and on the lipid profile in alloxan induced diabetic rats, thus signifying its efficacy as a prospective intake in diabetic complications.

## Materials and Methods

### Plant material

Fresh and flawless inflorescence of *Musa* sp. cv. Nanjangud rasa bale were collected from the nurturing farms of Nanjangud, Karnataka, India at coordinates 12.11° 7' 11" North, 76.70° 40' 58" East. The variety was identified and authenticated by the Department of Horticulture, Government of Karnataka, Mysore, India. Flowers were separated from the inflorescence and the spathe was discarded. The isolated flowers were cleaned, cut into small pieces and dried at 40°C in an oven. This was powdered using a homogenizer and further stored at 4°C until use.

### Preparation of extract and isolation of active compounds

The coarse powder was subjected to hot solvent extraction using ethanol in a Soxhlet apparatus. The extraction was performed twice with ethanol (500 ml) followed by filtration. The filtrate thus obtained was concentrated *in vacuo* using rotary evaporator (Rotavapor R-200, Buchi, Switzerland). The active compounds present in the ethanol extract of banana flower (EF) were identified as Umbelliferone (C1) and Lupeol (C2) using various spectroscopic methods *via* successive solvent extraction followed by repeated silica gel column chromatography [10].

### Animals

Healthy adult Wistar rats of either sex (equal number of male and female rats weighing 180–220 g) were obtained from the animal house of JSS College of Pharmacy, Ootacamund, India. The animals were housed in individual steel cages during the course of the experimental period in a room maintained at 25 ±1°C, with a 12 ± 1 h day and night schedule and were fed with standard rat feed and water *ad libitum*. The animals were acclimatized to the laboratory environment for 1 week before commencement of the experiment. The experiments were approved by Institutional Animal Ethics Committee (JSSCP/IAEC/Ph.D./PH.COLOGY/01/2013-14) and conducted as per the guidelines of CPCSEA, Chennai, India.

### *In vivo* Toxicity study

Healthy Wistar rats of either sex (equal number of male and female rats weighing 180–220 g) fasted for 12 hours were divided into drug-treated ‘test’ groups and vehicle-treated [1% CMC (Carboxy methyl Cellulose)] ‘control’ group, totally making up fourteen groups of six rats each. The ethanol extract of banana flower, Umbelliferone and Lupeol[250, 500, 1000, 2000 mg/kg body weight (b. wt.)] were separately administered to the rats in each of the test groups, while the control group was administered with 1% CMC, to evaluate the toxic effects produced on liver and kidney. Further the rats in both the test and control groups were provided access to food and water, and gross behavioural changes were observed over a period of 7 days for signs of acute toxicity [11].

### Antidiabetic activity of EF, Umbelliferone and Lupeol

#### Induction of diabetes

Diabetes was induced in overnight fasted rats weighing 180–220 g by a single intra peritoneal (i.p.) injection of 120 mg/kg alloxan monohydrate dissolved in freshly prepared saline. Animals with fasting blood glucose over 200mg/dl, 72 h after alloxan injection, were considered diabetic and such animals exhibiting uniform diabetic status were used for further studies. The treatment was initiated on the fourth day after alloxan injection, which was considered as the first day of treatment.

#### Experimental Design

Seven groups of six animals each were segregated for the study as indicated. Group 1:served as normal control (1% CMC alone); Group II: diabetic control (1% CMC alone); Group III: diabetic rats + 100 mg/kg b. wt. of EF in 1% CMC; Group IV: diabetic rats +200 mg/kg b. wt. of EF in 1% CMC; Group V: diabetic rats +100 mg/kg b. wt. of Umbelliferone in 1% CMC; Group VI: diabetic rats +100 mg/kg b. wt. of Lupeol in 1% CMC; Group VII: diabetic rats +250 mg/kg b. wt. of metformin in 1% CMC

The treatment was carried out up to 28 days. EF, Umbelliferone, Lupeol and metformin were dissolved in 1% CMC and administered orally (once daily) by gastric incubation with a force feeding (gauge) needle in a final volume of 1ml. Fasting blood glucose levels were checked every alternate week using Accu-Chek Blood Glucose Meters (Roche Diagnostics Pvt. Ltd, Mumbai, India). Urine sugar was assessed in the urine samples collected under a layer of toluene by 3, 5-dinitrosalycilic acid method [12]. The body weight, water intake and dietary intake were observed on day 0, 7, 14, 21 and 28 to evaluate the prevalence of improvement in the condition of polyphagia and polydipsia. These parameters were assessed by shifting rats to the metabolic cages for a period of 24 h. Following the treatment period (i.e. 28 days), the overnight fasted rats were anesthetized using pentobarbitone (30 mg/kg i.p.) and sacrificed by cervical decapitation during the early hours (between 8:00–9:00 am). The blood was drawn either from the retro orbital plexus (under mild anesthesia) during the experiment or from the heart at the time of sacrificing the rats after overnight fasting. It was collected in tubes with/without sodium heparin (20 U/ml blood, in 0.9% saline) for the plasma/ serum separation respectively, followed by centrifugation (2000 rpm for 15 minutes at 40°C). Liver was immediately exercised, washed with chilled physiological saline, homogenised (10% w/v) with 0.1 M Tris-HCl buffer (pH 7.4) and centrifuged (3000 rpm for 15 minutes at 40°C). The subsequent supernatant was quantified and used for various enzyme assays. Simultaneously, pancreas were dissected from the sacrificed animals (one from each group and 3 samples from each pancreas), cut into smaller pieces (around 1 mm X 1 mm X 1 mm), fixed with formalin solution (10%) and instantaneously processed for histopathological studies by paraffin method [13]. Briefly, the sections were processed by passing through different mixtures of ethyl alcohol and water (45, 75, 95% and finally incubated in alcohol) for dehydration, cleared in xylene and embedded in paraffin. Further, 5 μ sections of the tissues were obtained using rotary microtome, stained using hematoxylin–eosin (HME) dye and mounted in deparaffinised xylene medium for microscopic observation [13]. Micrographs (Canon 12.1 mega pixel digital camera, Japan) were captured using Axiostar plus microscope (Zeiss- Germany).

### Biochemical analysis

On the 0 and 28^th^day, blood was drawn and the haemoglobin content was evaluated by cyanmethaemoglobin method as described by David and Harold [14], and glycated haemoglobin by the method of Nayak and Pattabiraman [15]. Further, plasma insulin level was evaluated using DRG Insulin enzyme immunoassay kit (DRG diagnostics, Marburg, Germany) and protein content was estimated by Lowry method [16]. The separated serum was assayed for urea, creatinine and uric acid using commercially available diagnostics kit (Span Diagnostics Limited, Surat, India). Subsequently, serum aspartate aminotransferase (AST), alanine aminotransferase (ALT) and alkaline phosphatase (ALP) were analysed using diagnostics kit (Span Diagnostics Limited, Surat, India). Hepatic glycogen content and activities of glycogen synthase and glycogen phosphorylase were determined according to the methods of Kemp and Adrienne[17], Leloir and Goldemberg[18] and Cornblath et al.[19] respectively.

The activities of hepatic carbohydrate metabolic enzymes *viz*., Glucose-6-phosphate dehydrogenase (EC 1.1.1.49), Fructose-1, 6-bisphosphatase (EC 3.1.3.11) and Glucose-6-phosphatase (EC 3.1.3.9) were performed according to the method described by Raju et al. [20] Hexokinase (EC 2.7.1.1) assay was performed by the procedures suggested by Mahmood et al. [21]. Pyruvate kinase and lactate dehydrogenase activities were measured by the methods of King [22] and Pogson and Denton[23] respectively.

Plasma lipid profile such as triacylglycerol (TG), total cholesterol (TC) and high-density lipoprotein-cholesterol (HDL-C) levels were determined by enzymatic methods, using commercially available kits (Span Diagnostics Limited, Surat, India). The low-density lipoprotein-cholesterol (LDL-C) fraction, very low density lipoprotein-C (VLDL-C) and atherogenic index (AI) were calculated according to the formula below as described by William et al.[24]

LDL-C = TC – (Triglycerides/5+ HDL-C)

VLDL-C = Triglycerides/5

AI = (TC- HDL-C)/HDL-C

HTR (%) = HDL-C/TC ratio

The activities of enzymatic antioxidants *viz*., superoxide dismutase (SOD), glutathione peroxidase (GPx) and catalase (CAT) were assessed according to the method of Stefan and Gudrun[25], Rotruck et al.[26] and Asru[27] respectively. Glutathione (GSH) was assessed by Ellman's method [28]. The non-enzymatic antioxidants alpha-tocopherol and ascorbic acid were assessed following the methods of Baker et al.[29] and Stanley et al.[30]. Lipid peroxidation (LPO) was estimated colorimetrically by using thiobarbituric acid (TBA) reaction with malondialdehyde (MDA) as described by Hiroshi et al.[31].

### Statistical analysis

Results are expressed as Mean±SD. Statistical comparisons between normal and the treatment groups were performed by one-way analysis of variance (ANOVA), followed by Duncan’s Multiple Range Test using SPSS Software (version 21.0, Chicago, USA). The results were considered statistically significant if the ‘p’ values were 0.05 or less.

## Results

### Acute toxicity study

Oral administration of EF, C1 and C2 to animals up to a dose of 2000 mg/kg b. wt. did not exhibit any amendment in their behavioural pattern, physical parameters and no animal was found dead up to 7 days. In addition, at a dose of 2000 mg/kg b.wt., there was no substantial change in the body weight, water and food intake of the test animals in comparison to the control animals. Further, the animals neither produced any signs of toxicity nor mortality symptoms, thus illustrating the non-toxic nature of EF, C1 and C2. Therefore, further investigation of hypoglycaemic activity was carried out using 100 and 200 mg/kg dose levels.

### Antidiabetic action of EF and its isolated compounds

**Fig 1:**
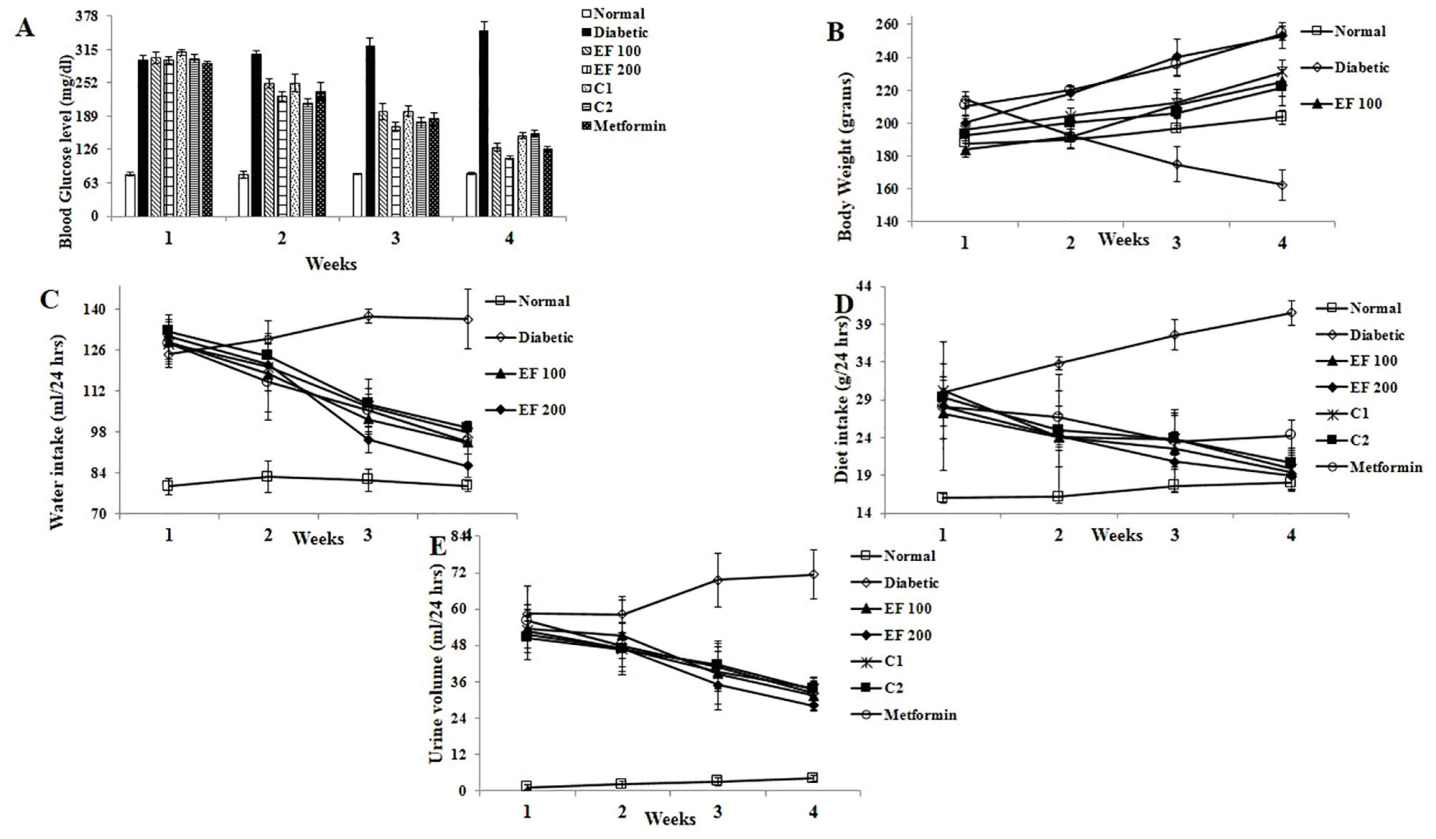
Effect of ethanol extract of flower (EF) and its isolated compounds on (A) fasting blood glucose levels (mg/dl), (B) body weight (grams), (C) water intake (ml/24 hours), (D) diet intake (grams/24 hours) and (E) urine volume (ml/24 hours) in control and diabetic rats. Parameters were monitored at weekly intervals (after 0, 14, 21, 28 days of treatment). Normal: normal control; diabetic: diabetic control; EF 100: Diabetic + EF (100 mg/kg b. wt.); EF 200: Diabetic + EF (200 mg/kg b. wt.); C1: Diabetic + Umbelliferone (100 mg/kg b. wt.); C2: Diabetic + Lupeol (100 mg/kg b. wt.); metformin: Diabetic + metformin (250 mg/kg b. wt.). Values are expressed as mean ± SD with six animals per group (n = 6).

As shown in Fig 1A, the fasting blood glucose (FBG) level of untreated diabetic rats (group 2) was significantly higher than normal control rats (group 1). Prior to the treatment with EF, C1 and C2, the FBG levels of diabetic groups (group 2–7) were almost similar (> 280 mg/dl). Treatment of diabetic rats with EF (100 and 200 mg/kg b.wt.), C1 and C2 (100 mg/kg b.wt.) ameliorated hyperglycaemia in a dose dependent decline-fashion for a period of 28 days, the reduction being 56.65%, 62.60%, 50.80% and 47.51% respectively, in FBG level when compared to the diabetic control rats whereas, metformin at 250 mg/kg b.wt. exhibited 55.88% reduction in FBG at the end of 28 days.

The baseline body weight of all the experimental group of rats initially was similar. At the end of the experimental period, the body weight of diabetic control rats was significantly reduced in contrast to the control group of rats (Fig 1B). While, oral administration of EF(100&200 mg/kg b. wt.), C1 (100 mg/kg b. wt.) and C2 (100 mg/kg b. wt.) to the diabetic rats enhanced (22.68%, 26.52%, 17.77% and 15.11% respectively) the body weight during the course of the experiment, metformin exhibited 20.99% increase in the body weight after the treatment.

As shown in Fig 1C–1E, the prevalence of diabetic symptoms *viz*., polydipsia, polyphagia and polyuria were observed in all diabetic groups of rats (group 2–7) after the injection of alloxan compared with normal control group of rats. At the end of the experiment (28 days), the water intake of diabetic rats reduced gradually by 26.32%, 30.22%, 24.70% and 23.47% for EF (100 and 200 mg/kg b.wt.), C1 and C2 (100 mg/kg b.wt.) treated groups respectively (Fig 1C). Furthermore, diabetic treated rats had a reduced diet intake at the end of 4 weeks (Fig 1D). The polyuria state of the rats treated with EF 100, EF 200, C1 and C2 (4.57%, 4.97%, 3.61% and 3.38% respectively) dropped gradually throughout the study in comparison to diabetic control rats (Fig 1E). To be precise, treatment with EF (100 & 200 mg/kg b.wt.), C1 and C2 (100 mg/kg b.wt.) to diabetic rats ameliorated the diabetic symptoms all through the study (28 days) in comparison to the control group of rats.

Metabolites excreted in urine such as sugar, protein, urea and creatinine were assessed on initial (day 0) and at the end of the experiment (day 28). Increased excretion of urine in case of diabetic rats persisted all through the experimental period and a similar trend was noticed in the urinary glucose excretion. Administration of EF (100 & 200 mg/kg b.wt.), C1 and C2 (100 mg/kg b.wt.) to diabetes induced rats exhibited a significant drop (-60.04%, -65.48%, -57.34% and -56.37% respectively) in glucosuria at the end of the study, compared to the initial values as observed in the beginning of the experiment, as well as the diabetic control group of rats (Table 1).

**Table 1:** Effect of ethanol extract of flower (EF) and its isolated compounds on urinary excretion of (a) glucose, protein, urea and creatinine (mg/24h respectively) and (b) serum protein, urea and creatinine (mg/24h respectively) in normal and experimental diabetic rats.

Sample			Group 1 (normal)	Group 2 (diabetic)	Group 3 (EF 100)	Group 4 (EF 200)	Group 5 (C1)	Group 6 (C2)	Group 7 (metformin)
**Urine**	**Glucose (mg/24h)**	**Day 0**	3.17±1.19^a^	7867.93±9.20^f^	7073.97±14.08^c^	7470.92±4.07^d^	7654.11±15.21^e^	6971.67±12.06^b^	7652.77±16.47^e^
		**Day 28**	2.59±6.69^a^	9112.83±2.31^g^	2827.19±15.08^c^	2757.54±7.62^b^	3265.50±11.40^f^	3042.27±13.81^d^	3072.44±13.14^e^
	**Protein (mg/24h)**	**Day 0**	3.51±1.07^a^	24.80±3.31^c, d^	21.65±1.50^b^	23.51±1.32^b, c, d^	22.13±2.37^c^	25.22±1.59^d^	21.31±3.73^b^
		**Day 28**	3.83±0.92^a^	28.50±2.19^d^	10.41±1.35^b^	9.46±1.16^b^	10.93±2.35^b^	12.98±1.94^c^	10.34±0.83^b^
	**Urea (mg/24h)**	**Day 0**	25.34±1.16^a^	246.47±4.67^e^	230.15±5.93^d^	244.77±2.66^e^	224.83±2.74^c^	213.26±2.94^b^	221.31±3.73^c^
		**Day 28**	26.09±1.03^a^	328.50±2.19^e^	132.58±3.31^c^	121.83±6.45^b^	139.56±4.32^d^	142.77±5.57^d^	122.00±4.00^b^
	**Creatinine (mg/24h)**	**Day 0**	6.08±2.01^a^	31.19±2.80^b^	30.80±3.53^b^	32.04±4.13^b^	28.24±3.83^b^	31.55±3.90^b^	31.23±2.79^b^
		**Day 28**	6.09±2.44^a^	39.49±3.62^d^	19.99±2.74^c^	15.08±3.47^b^	19.61±3.36^c^	20.23±3.81^c^	18.67±3.77^b, c^
**Serum**	**Protein (mg/24h)**	**Day 0**	7.45±2.61^b^	5.27±1.62^a^	4.87±1.55^a^	5.08±1.05^a^	5.26±0.81^a^	5.36±1.19^a^	5.40±0.67^a^
		**Day 28**	7.97±2.72^b^	4.28±1.22^a^	6.05±1.11^b^	6.87±1.47^b^	6.39±1.18^b^	6.39±1.20^b^	6.66±1.50^b^
	**Urea (mg/24h)**	**Day 0**	28.62±3.11^a^	54.93±8.19^b^	52.64±5.45^b^	52.04±5.80^b^	52.00±3.35^b^	49.59±4.30^b^	50.33±3.98^b^
		**Day 28**	29.71±2.01^a^	63.16±5.58^c^	39.04±2.05^b^	36.50±2.26^b^	39.83±5.60^b^	39.17±1.47^b^	37.67±4.68^b^
	**Creatinine (mg/24h)**	**Day 0**	0.43±0.11^a^	0.96±0.11^b^	0.93±0.09^b^	0.98±0.10^b^	0.95±0.06^b^	0.92±0.08^b^	0.98±0.12^b^
		**Day 28**	0.45±0.21^a^	1.42±0.14^c^	0.67±0.12^b^	0.68±0.11^b^	0.70±0.12^b^	0.69±0.06^b^	0.71±0.17^b^

Values are expressed as mean ± SD with six animals per group (n = 6). Means in the same row with distinct superscripts (a-f) are significantly different (p ≤0.05) as separated by Duncan multiple range test. Abbreviations and ‘n’ values are as defined in Fig 1.

It is clear from Table 1 that the normal control group of rats excreted a lower protein, urea and creatinine in urine compared to the diabetic control group of rats. Although oral administration of EF (100& 200 mg/kg b.wt.), C1, C2 (100 mg/kg b.wt. respectively) and metformin (250 mg/kg b.wt.) to diabetic rats for 28 days considerably reduced urine protein (-54.29%, -61.13%, -50.91%, -48.97% and -52.30% respectively), urea (-42.42%, -45.21%, -38.07%, -32.47% and -44.94% respectively) and creatinine levels to 35.99%, 52.99%, 30.82%, 36.52% and 40.50% respectively from 0 to 28 day, the values however remained significantly higher in comparison to the normal group of rats. Precisely, diabetic rats exhibited ~ two-fold decrease in serum protein and ~ two-fold increase in the serum urea and creatinine, compared to the normal control group of rats (Table 1). Oral administration of EF and its compounds for 28 days to diabetes induced rats enhanced the serum protein to near normal values besides reducing the serum urea and creatinine levels in comparison to the diabetic control group of rats.

### Improved biochemical parameters

**Fig 2:**
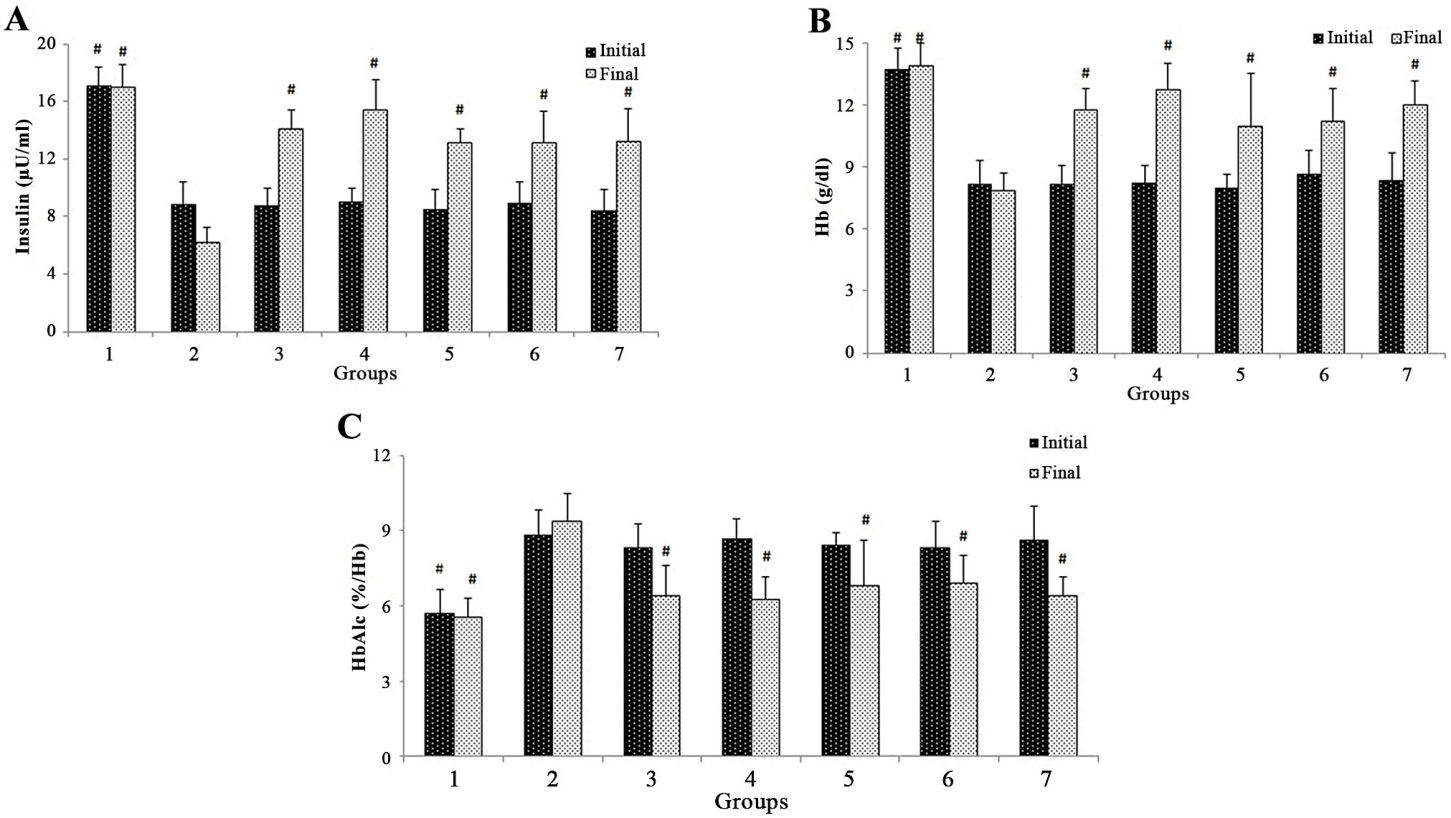
Effect of ethanol extract of flower (EF) and its isolated compounds on levels of (A)plasma insulin,(B) hemoglobin (Hb) and(C)glycated hemoglobin (HbA1c) of normal and experimental diabetic rats after 0 (initial) and 28 (final) days of treatment. Values are expressed as mean ± SD. Abbreviations and ‘n’ values are as defined in Fig 1.^**#**^significantly different (p ≤0.05) from diabetic control group.

At the beginning of the study, a significant drop of plasma insulin and Hb levels was observed in the case of diabetic induced rats compared to non-diabetic rats. Oral administration of EF and its constituents for 28 days enhanced the plasma insulin levels (Fig 2A) to 38.21%, 42.71%, 35.46% and 32.23% and Hb levels (Fig 2B) to 32.82%, 37.54%, 27.32% and 23.73% for EF (100& 200 mg/kg b.wt.), C1 and C2 (100 mg/kg b.wt.) respectively at the end of the study. Conversely, a higher level of HbA1c (%) was apparent in the alloxan-induced diabetic rats compared to normal control rats. Treatment of diabetic rats with EF at two doses *viz*., 100 & 200 mg/kg b.wt. and C1, C2 at 100 mg/kg b.wt. respectively for a period of 28 days is observed to have brought down the higher levels of HbA1c (%) to near normal at the end of the study (Fig 2C). A similar trend was witnessed with metformin drug fed at 250 mg/kg b.wt. to diabetic rats with decreased Hb, plasma insulin and an increase in HbA1c (%) levels.

Following the treatment with EF and its constituents for 28 days, the activities of serum AST, ALT and ALP were assessed both in normal and alloxan-induced diabetic rats. Untreated diabetic rats resulted with 2–3 fold increase in activities of AST, ALT and ALP in comparison to normal control rats (Table 2a). However, diabetic rats administered with the extract and isolated compounds for 4 weeks showed a marked reduction in activities when compared to untreated diabetic control rats.

**Table 2:** Effect of ethanol extract of flower (EF) and its isolated compounds on activities of (a) serum AST, ALT and ALP, (b) hepatic glycogen content, activities of glycogen synthase and glycogen phosphorylase and (c) activities of hepatic key enzymes of carbohydrate metabolism in normal and experimental diabetic rats after 28 days of treatment.

Estimates		Group 1 (normal)	Group 2 (diabetic)	Group 3 (EF 100)	Group 4 (EF 200)	Group 5 (C1)	Group 6 (C2)	Group 7 (metformin)
**a**	**AST**^1^*	28.84±2.32^a^	103.31±3.43^d^	41.92±5.00^b^	31.04±4.05^a^	49.59±1.99^c^	51.78±9.01^c^	48.65±3.78^c^
	**ALT**^1^	11.94±1.62^a^	21.93±2.77^c^	14.49±1.98^a^	13.26±3.07^a^	17.76±1.86^b^	18.94±1.37^b^	14.54±1.77^a^
	**ALP**^1^	31.21±3.19^a^	60.67±7.76^c^	34.17±4.71^a, b^	31.33±3.01^a^	39.83±7.41^b^	41.67±7.81^b^	38.33±6.05^a, b^
**b**	**Glycogen** ^2^	52.70±3.03^e^	16.99±1.43^a^	41.32±3.16^c^	47.37±2.70^d^	37.56±6.24^b, c^	33.88±1.74^b^	41.33±1.97^c^
	**Glycogen synthase** ^3^	805.81±5.61^f^	477.87±9.26^a^	742.54±8.40^d^	775.73±12.64^e^	730.58±8.12^c, d^	714.34±7.81^b^	728.36±20.03^c^
	**Glycogen phosphorylase** ^4^	608.50±9.48^a^	809.0±10.04^f^	709.17±9.26^d^	659.0±12.74^b^	718.0±8.49^d, e^	727.67±18.62^e^	693.56±16.06^c^
**c**	**Hexokinase** ^5^	0.26±0.02^d^	0.09±0.03^a^	0.24±0.05^c, d^	0.26±0.04^d^	0.20±0.01^b^	0.20±0.02^b^	0.21±0.04^b, c^
	**Glucose-6-phosphotase** ^4^	0.14±0.08^a^	0.33±0.06^b^	0.17±0.08^a^	0.16±0.07^a^	0.19±0.09^a^	0.21±0.09^a^	0.18±0.11^a^
	**Fructose-1,6-bisphosphotase**^4^	0.07±0.05^a^	0.26±0.08^c^	0.13±0.04^a, b^	0.10±0.06^a, b^	0.14±0.04^b^	0.15±0.02^b^	0.11±0.02^a, b^
	**Glucose-6-phosphate dehydrogenase** ^6^	3.40±.09^f^	2.21±0.08^a^	3.03±0.05^d^	3.19±0.03^e^	2.88±0.08^b, c^	2.93±0.09^c^	2.80±0.10^b^
	**Lactate dehydrogenase** ^7^	242.38±10.50^a^	454.31±6.68^e^	279.71±3.60^c^	267.55±7.49^b^	283.90±9.00^c, d^	290.77±6.71^d^	270.54±8.64^b^
	**Pyruvate kinase** ^7^	187.80±7.47^d^	88.58±5.50^a^	154.23±4.36^b^	163.19±6.57^c^	150.15±14.05^b^	145.85±4.17^b^	149.01±4.95^b^

AST: aspartate aminotransferase; ALT: alanine aminotransferase; ALP: alkaline phosphatase.

*^(1)^:IU/L

^(2)^: mg/g tissue

^(3)^:μmoles of UDP formed/hour/mg protein

^(4)^: μmoles of Pi liberated/hour/mg protein

*^(5)^:μmoles of glucose phosphorylated/h/mg protein

^(6)^:U/mg protein

^(7)^: μmoles of Pyruvate formed/h/mg protein

Values are expressed as mean ± SD. Means in the same row with distinct superscripts (a-f) are significantly different (p ≤0.05) as separated by Duncan multiple range test. Abbreviations and ‘n’ values are as defined in Fig 1.

### Amelioration of hepatic/glucose metabolising enzymes

Diabetic state led to a significant decrease in hepatic glycogen levels, in consort with a reduction in the glycogen synthase activity and an increase in the activity of glycogen phosphorylase (Table 2b). The diabetic treated group of rats (group 3–7) exhibited enhanced activity of glycogen synthase and hepatic glycogen levels, in addition to a declined level of glycogen phosphorylase activity compared to those of diabetic control group of rats.

Table 2c **depicts** the effect of EF and its constituents on carbohydrate metabolising enzymes in the liver of normal/diabetic control and treated group of rats. It is clear that untreated diabetic control rats exhibited a lesser level of activities of hexokinase, glucose-6-phosphate dehydrogenase and pyruvate kinase, but enhanced activities of gluconeogenic enzymes (glucose-6-phosphatase, fructose-1,6-bisphosphatase and lactate dehydrogenase) in the liver than those of normal control rats. All the diabetic rats treated with EF and its constituents for a period of 4 weeks reversed the levels of gluconeogenic enzymes as well as the activities of key enzymes of carbohydrate metabolising enzymes to near normal when compared to normal control rats.

### Attenuation of lipid profile

The results illustrated that, in alloxan induced untreated diabetic rats the serum lipid profile *viz*., TC, TG, LDL-C and VLDL-C was increased and HDL level was decreased in comparison to the normal control rats. However, diabetic rats treated with EF and its constituents exhibited a marked reversal of the serum lipid profile compared to the untreated diabetic group of rats. In addition, a decrease in HTR (%) and a corresponding increase in AI, LDL-C/HDL-C ratio, free fatty acids and phospholipids are evident in diabetic state. These were ameliorated by the effect of EF and its compounds to a striking amount when compared to the diabetic control group of rats at the end of the study (Table 3a).

**Table 3:** Effect of ethanol extract of flower (EF) and its isolated compounds on (a) serum and (b) liver lipid profile in normal and experimental diabetic rats after 28 days of treatment.

Estimates		Group 1 (normal)	Group 2 (diabetic)	Group 3 (EF 100)	Group 4 (EF 200)	Group 5 (C1)	Group 6 (C2)	Group 7 (metformin)
**a**	**Total cholesterol** ^w^*	71.52±3.61^a^	137.75±5.82^d^	79.94±4.82^b, c^	74.56±4.81^b, c^	80.13±4.53^b, c^	84.09±3.99^c^	79.91±4.04^b, c^
	**Triglycerides** ^w^	45.96±7.88^a^	107.0±5.50^e^	60.86±2.61^c, d^	54.44±6.80^b^	65.18±2.73^d^	67.18±5.34^d^	58.25±2.67^b, c^
	**HDL-C** ^w^	29.30±5.95^d^	15.04±4.39^a^	24.03±3.69^c, d^	26.36±5.09^c, d^	23.89±3.46^c, d^	21.53±4.25^b, c^	18.25±4.27^a, b^
	**LDL-C** ^w^	33.03±7.66^a^	101.30±8.04^d^	43.74±4.82^b, c^	37.31±7.93^a, b^	43.20±5.39^b, c^	49.12±5.60^c^	50.02±7.26^c^
	**VLDL-C** ^w^	9.13±1.58^a^	21.41±1.10^e^	12.17±0.52^c, d^	10.89±1.36^b^	13.04±0.55^d^	13.44±1.07^d^	11.65±0.53^b, c^
	**Atherogenic index** ^x^	1.53±0.55^a^	8.76±2.49^c^	2.39±0.53^a, b^	1.96±0.86^a^	2.41±0.52^a, b^	3.04±0.81^a, b^	3.62±1.27^b^
	**LDL-C/ HDL-C ratio**	1.21±0.50^a^	7.24±2.10^c^	1.88±0.45^a, b^	1.53±0.74^a^	1.86±0.46^a^,^b^	2.39±0.65^a, b^	2.95±1.08^b^
	**HTR (%)** ^y^	41.16±9.28^d^	10.97±3.41^a^	30.08±4.64^b, c^	35.65±7.80^c, d^	29.86±4.39^b, c^	25.69±5.57^b^	23.03±6.25^b^
	**Free fatty acids** ^w^	52.71±11.82^a^	101.40±11.16^b^	63.03±16.09^a^	55.48±8.30^a^	64.06±10.06^a^	63.94±9.44^a^	67.15±10.83^a^
	**Phospholipids** ^w^	77.71±7.63^a^	126.40±12.05^c^	90.60±10.61^a, b^	87.48±4.13^a, b^	92.70±14.49^b^	90.73±7.15^a, b^	98.82±12.45^b^
**b**	**Total cholesterol** ^z^	3.35±1.97^a^	6.92±2.96^b^	4.86±2.13^a, b^	4.20±2.09^a, b^	4.70±1.78^a, b^	5.36±2.10^a, b^	5.86±2.19^a, b^
	**Triglycerides** ^z^	2.56±1.56^a^	5.73±2.31^b^	3.80±1.69^a, b^	3.30±2.60^a, b^	4.13±1.74^a, b^	4.12±2.64^a, b^	4.46±1.68^a, b^
	**Free fatty acids** ^z^	8.17±2.55^a^	17.30±6.83^b^	8.63±3.13^a^	8.29±2.48^a^	8.30±3.93^a^	8.80±1.60^a^	9.63±4.42^a^
	**Phospholipids** ^z^	22.37±3.26^a^	43.71±11.03^b^	26.37±7.55^a^	24.21±8.53^a^	28.37±6.21^a^	30.54±7.19^a^	30.54±6.68^a^

*^(w)^: mg/dl

^(x)^: (TC-HDL)/HDL

^(y)^: HDL-C/TC ratio

^(z)^: mg/g of wet tissue

Values are expressed as mean ± SD. Means in the same row with distinct superscripts (a-e) are significantly different (p ≤0.05) as separated by Duncan multiple range test. Abbreviations and ‘n’ values are as defined in Fig 1.

Table 3b illustrates the levels of TC, TG, free fatty acids and phospholipids in the liver of control and diabetic rats. In comparison to the normal control rats, diabetic control rats showed almost two-fold increase in liver-lipid profile. Diabetic rats treated with the drugs exhibited a marked reduction in the liver-lipid profile in comparison to the diabetic control group of rats.

### Activities of enzymatic and non-enzymatic antioxidant levels

The enzymatic antioxidants and non-enzymatic antioxidants in serum and liver of normal and experimental rats after 28 days of treatment are as shown in Table 4. Diabetic untreated group of rats had nearly 3-fold increase of lipid peroxide content in both serum and liver when compared to normal control rats whereas, administration with EF and its compounds to diabetic induced rats reverted the increased levels to near normal. In diabetic control group, a significant reduction of enzymatic antioxidants *viz*., SOD, CAT and GPx were identified in serum (65.83%, 70.42% and 78.08%) and in liver (58.12%, 59.79% and 56.48%) respectively as compared to those of normal control group of rats. Treatment for 28 days with EF (100 & 200 mg/kg b.wt.), C1 (100 mg/kg b.wt.), C2 (100 mg/kg b.wt.) and metformin (250 mg/kg b.wt.) reverted these antioxidant levels in the serum and liver to near-normal. Besides, the non-enzymatic antioxidants of the untreated diabetic rats remained much lower than those of normal control rats, while a significant improvement in GSH, vitamin C and Vitamin E levels were observed in diabetic treated rats with EF, C1, C2 and metformin as compared to the normal control group of rats.

**Table 4:** Effect of ethanol extract of flower (EF) and its isolated compounds on lipid peroxidation, enzymatic and non-enzymatic antioxidants in (a) serum and (b) liver of normal and experimental diabetic rats after 28 days of treatment.

Estimates		Group 1 (normal)	Group 2 (diabetic)	Group 3 (EF 100)	Group 4 (EF 200)	Group 5 (C1)	Group 6 (C2)	Group 7 (metformin)
**a**	**Lipid peroxidation** ^v^*	0.26±0.07^a^	0.79±0.08^c^	0.45±0.09^b^	0.43±0.13^b^	0.47±0.08^b^	0.49±0.09^b^	0.47±0.08^b^
	**Superoxide dismutase**^w^	6.85±0.87^d^	2.90±1.04^a^	5.86±1.66^b, c, d^	6.62±1.82^c, d^	5.06±1.76^b, c^	4.41±1.07^a, b^	4.75±1.22^b^
	**Catalase** ^x^	3.68±1.18^c^	1.15±0.18^a^	2.59±0.65^b^	2.74±0.75^b^	2.39±0.92^b^	1.94±0.45^a, b^	2.61±0.67^b^
	**Glutathione peroxidase** ^y^	0.73±0.27^c^	0.16±0.07^a^	0.57±0.12^b, c^	0.63±0.13^b, c^	0.54±0.09^b^	0.49±0.05^b^	0.51±0.10^b^
	**Reduced glutathione** ^y^	3.11±0.87^b^	1.56±0.27^a^	2.43±0.38^a, b^	2.84±0.71^b^	2.68±1.00^b^	2.18±0.91^a, b^	2.16±0.66^a, b^
	**Vitamin C** ^z^	2.99±0.30^c^	1.98±0.51^a^	2.86±0.55^b, c^	3.03±0.35^c^	2.36±0.49^a, b^	2.72±0.51^b, c^	2.60±0.33^b, c^
	**Vitamin E** ^z^	1.72±0.20^b^	1.0±0.08^a^	1.52±0.44^b^	1.71±0.16^b^	1.62±0.17^b^	1.45±0.19^b^	1.57±0.02^b^
**b**	**Lipid peroxidation** ^v^*	4.36±0.99^a^	12.07±1.35^c^	9.32±1.25^b^	8.98±1.55^b^	9.82±1.84^b^	9.96±1.96^b^	9.94±1.21^b^
	**Superoxide dismutase** ^w^	39.18±2.43^e^	16.52±2.60^a^	34.18±3.20^c, d^	36.18±3.71^d, e^	30.85±3.29^b, c^	29.52±3.79^b^	29.68±1.97^b^
	**Catalase** ^x^	29.87±1.65^c^	12.53±1.12^a^	22.03±1.47^b^	22.90±1.72^b^	21.86±2.62^b^	21.19±1.12^b^	22.86±1.99^b^
	**Glutathione peroxidase** ^y^	6.42±0.86^b^	3.03±0.57^a^	4.94±1.57^b^	5.08±1.53^b^	5.01±1.48^b^	4.86±1.50^b^	5.10±1.46^b^
	**Reduced glutathione** ^y^	18.32±1.32^d^	11.33±1.11^a^	14.83±1.30^b, c^	15.83±1.97^c^	15.16±1.04^b, c^	14.66±0.90^b, c^	13.99±0.96^b^
	**Vitamin C** ^z^	1.52±0.18^b^	0.99±0.15^a^	1.45±0.43^b^	1.58±0.34^b^	1.54±0.37^b^	1.51±0.39^b^	1.31±0.08^a, b^
	**Vitamin E** ^z^	0.81±0.12^b^	0.51±0.10^a^	0.69±0.24^b^	0.78±0.12^b^	0.72±0.10^b^	0.70±0.08^b^	0.70±0.09^b^

*^(v)^: μmol of MDA/mg protein

^(w)^: Units of activity/mg protein

^(x)^: μmol of H_2_O_2_/min/mg protein

^(y)^: μmol of NADPH/min/mg protein

^(z)^: Units/mg protein

Values are expressed as mean ± SD. Means in the same row with distinct superscripts (a-e) are significantly different (p ≤0.05) as separated by Duncan multiple range test. Abbreviations and ‘n’ values are as defined in Fig 1.

In general, the treatment to alloxan induced diabetic rats with EF at two doses *viz*., 100 and 200 mg/kg b.wt. and C1 and C2 at 100 mg/kg b.wt. respectively for a period of 28 days illustrated a significant effect for all the biochemical parameters studied above in comparison with the control rats. Of all the doses, EF at 200 mg/kg b.wt. revealed the maximum effect which was significantly higher (p <0.05) than metformin (250 mg/kg b.wt.). Further, it was observed that Umbelliferone and Lupeol also brought back all these parameters to near normal and the values were comparable to those of metformin.

### Histology of pancreas

**Fig 3:**
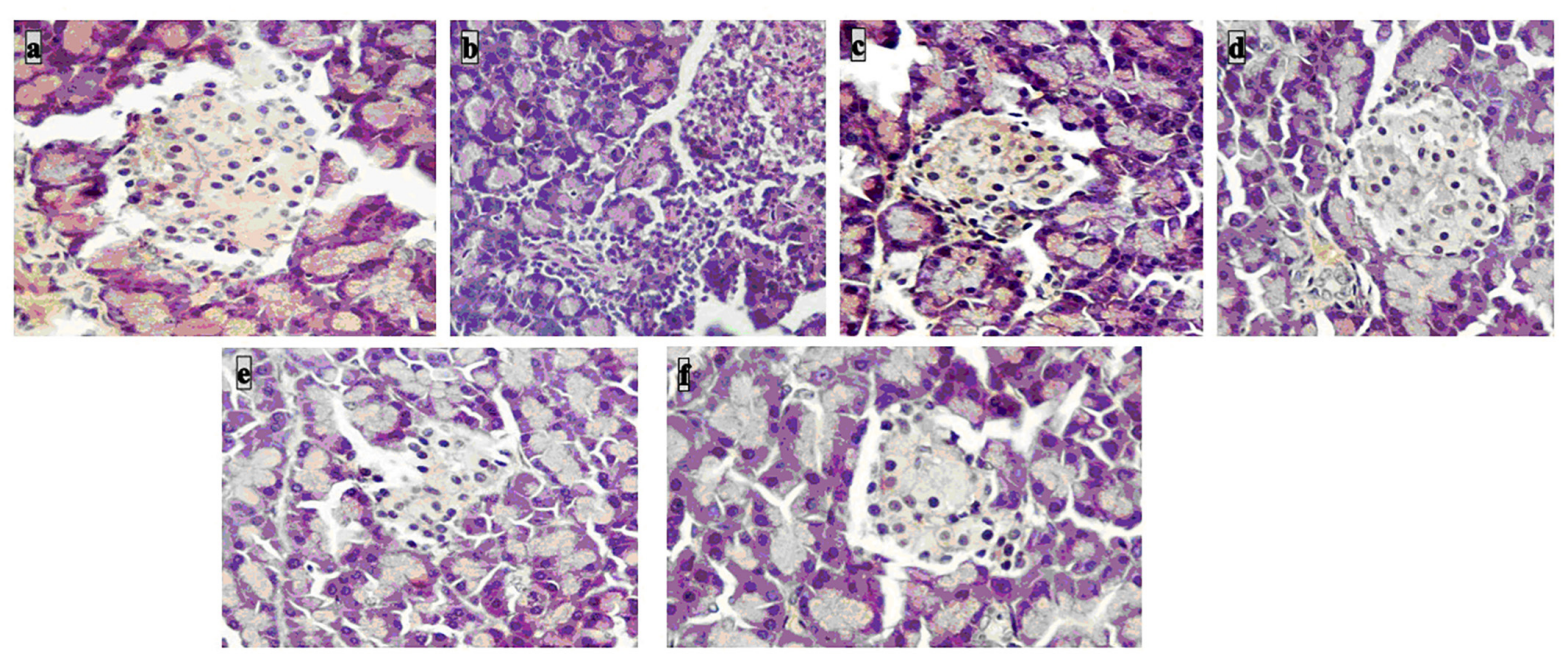
Histopathological examinations of pancreas in experimental rats after 28 days of treatment with ethanol extract of flower (EF) and its isolated compounds (H and E, 40 x). Micrographs showing normal **(a)** intact islets with cellular characteristics of pancreas; diabetic group **(b)** with degeneration of pancreatic acini, apathy of β cells (necrotic and fibrotic changes) and emaciated islets of Langerhans; EF 100 **(c)**, EF 200 **(d)**, C1 **(e)**, C2 **(f)** and metformin **(g)** restored normal cellular population of pancreatic acini/lobules and with size, shape of the islets.

Histopathological results gave steady confirmation to all the biochemical parameters studied. Histological examination of the sections of pancreas illustrated that the normal control group of rats had normal pancreatic islets with exocrine acini and endocrine cells massed with cytoplasm (Fig 3a), whereas dilated and de-granulated islet cells were observed in the untreated diabetic group of rats (Fig 3b). The diabetic rats treated with the drugs refurbished the changes and had nearly normal architecture with non-attendance of dilation and granulated cells presenting hyperplasticity of islets (Fig 3c–3e).

## Discussion

While hyperglycaemic condition is known for its increased risk of cardiovascular disorders due to its direct implications on the blood lipid profiles, it is also held responsible for several other disorders collectively known as ‘diabetic complications’ [3]. Traditional medical practises have advocated the control of several disorders by dietary modulation which undeniably is the best approach even for the management of diabetes. Fruits and vegetables which constitute rich sources of several bioactive ingredients have been advantageous while consumed in the right quantity [2]. It is the banana flower (a by-product of banana cultivation), which is consumed as vegetable in many countries that is being taken up for our investigation. Its vast ethnomedical history and rich phytochemical reservoir of heuristic medicinal value has provided a basis for further studies on its beneficiary aspects [32].

Our previous investigation [10] showing the potent *in vitro* antihyperglycaemic and antioxidant activity of EF and its constituents Umbelliferone and Lupeol, asserted the ethnomedical use of this against diabetes. In the present study, we investigated whether the EF and its constituents had any *in vivo* effect of hypoglycaemic, hypolipidaemic, and antioxidant action in alloxan-induced diabetic rats. Alloxan treatment is known to induce diabetes by a massive destruction of the β-cells of the islets of langerhans which creates an insulin-deficient state leading to prolonged hyperglycaemia [33]. β-cells of the islets rapidly take up alloxan leading to a transient raise in the insulin levels. This is followed by the generation of ROS which are known to fragment the DNA of the pancreatic islets, thus leading to an impaired pancreas with reduction in the insulin secretion [34]. In our study, the treatment of EF and its constituents to the diabetic rats have favoured the regeneration of the β-cells. The histopathological observations also exhibit an improved pancreatic β-cells and were similar to the control animals. Such a regeneration is hypothesized to be due to the significant antioxidant properties exhibited by the extract as reported in our previous *in vitro* study [10]. EF, C1 and C2 are proven to also possess remarkable free radical scavenging ability, which could also be the means for protection against alloxan toxicity in the diabetic rats. The improvement in the insulin secretion is thereby an effect of either or a constitutive effect of both the mechanisms [35].

Further, in the present investigation EF, C1 and C2 significantly reduced the plasma glucose levels (in groups receiving 200 mg/kg b.wt. of EF followed by 100 mg/kg b.wt. of EF, C1 and C2) along with a corresponding increase in the insulin levels. The observed hypoglycaemic effect may be due to either regeneration of β-cells or enhanced insulin sensitivity to the target tissues. Further, to assert the mode of action, the insulin levels were estimated along with the histology of the pancreas. The diabetic rats showing reduced insulin levels and the degranulated and dilated islets were restored after treatment with EF, C1, C2 and metformin. Recent studies [36–37] have reported similar effects on the pancreatic β-cell stimulation using several isolated compounds which are concordant to our present findings.

Diabetes is linked to polydipsia, polyphagia and polyuria which are also exhibited in the alloxan-induced diabetic model rats. During glucose uptake by the cells, due to the osmotic effect, water is also carried into the cells which in turn activate the thirst centres of the brain leading to polydipsia and polyuria [38]. Similarly, the impeded regulation of glucose storage pathway createsincreased glucose levels in the circulation. However, since this glucose is not available to the tissues due to the insulin deficiency, there exists a constant thirst for glucose in the tissues. This in turn, leads to the breakdown of structural proteins to replenish amino acids, which are further converted to glucose *via* the gluconeogenic pathway [39]. Owing to this impairment, an unintentional weight loss because of the proteolysis of the structural proteins is caused as a characteristic in diabetic patients. The increased food and water consumption exhibited by the alloxan induced diabetic rats were reversed as a result of the administration of EF and its constituents (C1 and C2), improving the glycaemic control and regeneration of β-cells to recover normal insulin levels. It can thus be suggested that EF and its constituents (C1 and C2) assist in the management of these complications of diabetes.

Elevated glucose levels (glucose toxicity) led renal dysfunctioning is also a characteristic in diabetics. Owing to the persistently high levels of blood glucose and correspondingly high protein degradation, an elevated urea and creatinine levels are attributed to the impaired renal regulations [40]. EF, C1 and C2administration showed effective protection against the hyperglycaemia-induced renal alterations exhibiting a remarkable reduction in the activities of these markers of renal functioning. Of the complications caused by prolonged hyperglycaemia, the other major concern is its effects on the circulating and structural proteins. Apart from the excessive protein degradation leading to muscle wasting and weight loss, there is an increased risk of irreversible glycation on various proteins such as haemoglobin (Hb), albumin, collagen and low-density lipoprotein. HbA1c levels also constitute as an important marker of diabetes which remain high and reflect the level of glycaemic control during the condition [36]. It was evident from the study that after administration of EF and its constituents (C1 and C2), an optimal control of the glycaemic index was observed as reflected by the reduction in the levels of HbA1c and a concomitant increase in the levels of Hb.

Apart from the renal impairment, liver is also affected by the glucose toxicity induced by high levels of glucose for an extended period of time. Liver being the primary organ for the glucose storage and metabolism, it is severely affected during diabetic conditions [41]. Glucose metabolism is intricately regulated by different pathways of its storage and degradation. Normally, excess glucose is stored in the form of glycogen by glycogenesis pathway of which, glycogen synthase constitutes the key regulatory enzyme [42]. While, the blood glucose levels are maintained by their storage in liver, the same is also achieved by the increased breakdown of glucose by the glycolytic pathway. An insulin-dependent hexokinase (which catalyzes the phosphorylation of glucose for its further breakdown), lactate dehydrogenase (which catalyzes the inter-conversion of lactate to pyruvate) and pyruvate kinase (which catalyzes the conversion of phosphoenol pyruvate) are the key regulatory enzymes of the pathway and deliver pyruvate for the subsequent citric acid cycle. There exists a marked decrease in the activitiesof these enzymes owing to the reduced insulin levels which are also the makers of diabetic state [43]. In our study, EF, C1 and C2 treatment facilitated the insulin production, in turn, leading to the enhanced activities of these regulatory enzymes which was responsible for the antihyperglycaemic effects and proved the beneficiary aspects of EF, C1 and C2 in this regard. This effect is witnessed probably due to the improvement in the insulin levels in the circulation that is exerted by the extracts and its constituents.

Despite the distinctive feature of diabetes being hyperglycaemia, glucose available to the tissues is limited. This state invites the activation of the gluconeogenic pathway in order to replenish glucose load to the tissues and for this, the key enzymes employed are glucose-6-phosphatase and fructose-1, 6-bisphosphatase which remain exceedingly high during the state of the disease [44]. Furthermore, glucose gains an important precursor for the nucleotide metabolism, by feeding pentose sugar in the form of ribose-5 phosphate via the pentose phosphate pathway (precursor: glucose) and the reducing equivalent NADPH via the hexoze monophosphate shunt pathway (precursor: intermediate of glucose hydrolysis). A state of oxidative damage is attained due to the relatively low levels (response to diabetes) of glucose-6-phosphate dehydrogenase which is the key enzyme in the synthesis of this pentose sugar (pentose phosphate pathway). Treatment with EF and the constituents (C1 and C2) assisted in gaining metabolic control over these enzymes in order to regulate the levels of glucose in the circulation. The gluconeogenic enzyme activities were dropped remarkably while improving the activities of the enzymes of pentose phosphate pathway, thus rendering a smooth glycaemic profile [20, 39]. These results suggest the potential of EF, C1 and C2 not only in reducing hyperglycaemia but also in management of other complications affecting the kidney and liver.

As markers of the liver functioning, AST, ALT and ALP enzyme activitiesat the basal range are elevated during diabetic state which signifies the toxic effect of alloxan on liver [36]. These are the enzymes responsible for the conversion of amino acids to keto acids and their levels could be elevated mainly because of the damage to the liver leading to their leakage into the blood streams. EF, C1 and C2 treatment has rendered a protective role by reducing the activities of these enzymes thus suggesting their hepatoprotective potential.

While the microvascular complications of diabetes are neuropathy, retinopathy and nephropathy, there are some macrovascular risks collectively known as cardiovascular complications. Insulin not only regulates the levels of glucose in blood but also maintains a smooth lipid profile by directing the expression of lipoprotein lipase enzyme responsible for the hydrolysis of triglycerides [45]. Insulin deficit during diabetes, thus results in the accumulation of dietary lipids leading to abnormalities such as hyperlipidaemia raising the risks factors for coronary heart disorders [46]. Diabetes is also characterized by high levels of TG, TC, LDL, VLDL and lower levels of HDL attributed to dyslipidaemia. Such a variation in the lipid profile is mainly due to the increased mobilization of fatty acids from the adipocytes to replenish glucose deficit in the peripheral tissues. Amelioration of the insulin secreting cells could have probably led to the improvement in the levels of lipoprotein lipases which was observed after the EF, C1 and C2 treatment leading to normal blood lipid profile. Also, the imbalanced HDL/LDL ratio caused by diabetes improved to a great extent by the EF treatment signifying its role in reduction of lipid accumulation in the tissues. Further, EF also improved the HDL-C or HTR ratio which is the primary character of any anti-atherogenic agent and is in agreement with several studies [2] that have demonstrated that the level of HDL-C is inversely proportional to the incidence of cardiovascular disorders. As an ancillary to these activities, the EF and its constituents may also possess effect on the bile acid synthesis and excretion which are known to be responsible for the improvement in the levels of TG and HDL-C.

Oxidative stress has been implicated in the pathophysiology of several diseases including diabetes and its related complications. Free radical generation/the mitochondrial superoxide production which is the reason for oxidative stress, is known to be a consequence of one or a few of the mechanisms *viz*., increased glycolysis and activation of polyol pathway, non-enzymatic protein glycation and auto-oxidation of the excessive glucose along with a drop in the tissue levels of the antioxidant enzymes [36]. Similarly, in the alloxan-induced diabetic rat models a marked depletion in the antioxidant enzymes was observed in our study, which answers the oxidative stress-mediated damage rendered to the serum and liver. The free radical scavenging system includes enzymatic (SOD, CAT and GPx) and non-enzymatic (GSH, vitamin C, vitamin E) antioxidants which are stringently regulated in normal conditions [46]. The levels of all these antioxidants dropped and a proportionally higher level of TBARS was witnessed due to the diabetic manifestation. In the present study, evaluation of the antioxidant potential (enzymatic) and levels of the oxidative stress markers (non-enzymatic) post-administration of EF apparently suggested an overwhelming amelioration thereby counteracting the damages caused to the tissues by oxidative stress. A further damage caused by the oxidative stress is the peroxide formation of the membrane lipids due to the oxidation by free radicals, resulting in membrane dysfunctioning [47–48]. Accumulation of such peroxides was found to have reduced to notable levels after administration of EF, C1 and C2signifying its potential in not only improving the microvascular but also macrovascular diabetic complications.

## Conclusion

Insulin is known to be the primary hormone responsible for the maintenance of blood glucose and lipid levels which consecutively control oxidative stress induced tissue damage. In the present study, the improvement in the β-cell structure and cell mass was witnessed after the EF, C1 and C2 treatment, suggesting its protective ability on the pancreatic cells. The resulting amelioration of insulin levels and the inhibitory potential of EF on the carbohydrate metabolizing enzymes had definitive effects thereby resulting in anti-hyperglycaemic, antilipidemic as well as antioxidant properties. These results are promising in considering EF and its constituents Umbelliferone and Lupeol as potential antidiabetic herbal remedies in the management of diabetes and its associated complications.
