# Correction: Assessment of *In Vivo* Antidiabetic Properties of Umbelliferone and Lupeol Constituents of Banana (*Musa* sp. var. Nanjangud Rasa Bale) Flower in Hyperglycaemic Rodent Model

**DOI:** 10.1371/journal.pone.0160048

**Published:** 2016-07-20

**Authors:** Ramith Ramu, Prithvi S. Shirahatti, Nanjunda Swamy S., Farhan Zameer, Dhananjaya BL, Nagendra Prasad M. N.

The fifth author’s name is presented incorrectly within the citation. The correct citation is: Dhananjaya BL. The correct citation is: Ramu R, S. Shirahatti P, S. NS, Zameer F, Dhananjaya BL, M. N. NP (2016) Assessment of *In Vivo* Antidiabetic Properties of Umbelliferone and Lupeol Constituents of Banana (*Musa* sp. var. Nanjangud Rasa Bale) Flower in Hyperglycaemic Rodent Model. PLoS ONE 11(3): e0151135. doi:10.1371/journal.pone.0151135
